# A Cluster Randomized Trial to Promote Healthy Menu Items for Children: The Kids’ Choice Restaurant Program

**DOI:** 10.3390/ijerph14121494

**Published:** 2017-12-01

**Authors:** Guadalupe X. Ayala, Iana A. Castro, Julie L. Pickrel, Shih-Fan Lin, Christine B. Williams, Hala Madanat, Hee-Jin Jun, Michelle Zive

**Affiliations:** 1 Graduate School of Public Health and the Institute for Behavioral and Community Health (IBACH), San Diego State University, 5500 Campanile Drive, San Diego, CA 92182, USA; hmadanat@mail.sdsu.edu; 2 Marketing Department, Fowler College of Business and IBACH, San Diego State University, 5500 Campanile Drive, San Diego, CA 92182, USA; iana.castro@mail.sdsu.edu; 3 IBACH, San Diego State University Research Foundation, 9245 Sky Park Court, Suite 220, San Diego, CA 92123, USA; jpickrel@mail.sdsu.edu (J.L.P.); slin@mail.sdsu.edu (S.-F.L.); hjun@mail.sdsu.edu (H.-J.J.); 4 Department of Pediatrics, University of California, San Diego, Family Medicine and Public Health, 9500 Gilman Drive, #0725, La Jolla, CA 92093, USA; cwilliams@ucsd.edu (C.B.W.); mzive@ucsd.edu (M.Z.)

**Keywords:** restaurant, food availability, food marketing, child menu

## Abstract

Evidence indicates that restaurant-based interventions have the potential to promote healthier purchasing and improve the nutrients consumed. This study adds to this body of research by reporting the results of a trial focused on promoting the sale of healthy child menu items in independently owned restaurants. Eight pair-matched restaurants that met the eligibility criteria were randomized to a menu-only versus a menu-plus intervention condition. Both of the conditions implemented new healthy child menu items and received support for implementation for eight weeks. The menu-plus condition also conducted a marketing campaign involving employee trainings and promotional materials. Process evaluation data captured intervention implementation. Sales of new and existing child menu items were tracked for 16 weeks. Results indicated that the interventions were implemented with moderate to high fidelity depending on the component. Sales of new healthy child menu items occurred immediately, but decreased during the post-intervention period in both conditions. Sales of existing child menu items demonstrated a time by condition effect with restaurants in the menu-plus condition observing significant decreases and menu-only restaurants observing significant increases in sales of existing child menu items. Additional efforts are needed to inform sustainable methods for improving access to healthy foods and beverages in restaurants.

## 1. Introduction

Away-from-home (AFH) eating remains a serious threat to children’s and adults’ risk for overweight and obesity [1]. This is a public health problem, with estimates suggesting that AFH eating accounts for 34% of daily calories that are consumed by children in the United States (U.S.) [2]. To address obesity at a population level, policymakers, practitioners, and researchers have called for creating health-promoting environments, including food environments that support healthy food and beverage choices [3]. This is particularly relevant in racial/ethnic and lower-income communities in the U.S. where “food swamps” (i.e., areas with few healthy options versus relatively large amounts of energy-dense snacks and beverages [4]), have been identified as a threat to the health of community members [5,6].

### 1.1. Diet, Obesity and the Restaurant Food Environment

Restaurants are an important target for intervention to promote healthy eating [7,8]. Recent evidence points to the lack of healthy menu items on child menus in national chain restaurants [9]. We found that most independent restaurants in rural environments did not have child menus [10], as compared with independent restaurants in neighbouring urban communities [11]. Notwithstanding, independent restaurants are important to target given evidence that 92% of restaurant meals exceeded energy requirements for a single eating occasion [12]. Our focus on sit-down (i.e., full-service) restaurants is important given the larger proportion of calories that are consumed from these types of restaurants versus fast food restaurants among children [13].

### 1.2. Evidence for Promoting Healthy Menu Items to Children and Families

Evidence suggests that restricted or guided choice restaurant-based interventions may be more effective than information only campaigns in promoting the sale of healthier options [14,15]. Evidence from studies in the United Kingdom, Australia, and other countries suggest the need for more evidence-based strategies, especially those that require minimal time and effort on the part of restaurant personnel (i.e., “health by stealth” page 228; [15]). However, evidence is more limited from research targeting child menus that involves independent restaurants [3,7]. Independent restaurants represent 66% of all restaurants in the U.S. [16], thus, they are an important segment of this market. Furthermore, ethnic food restaurants are more likely to be independent [16], and attract individuals with the greatest risk for health disparities in obesity [17]. However, unlike chain restaurants, they have fewer resources to test meaningful approaches to modifying their menus to promote healthier menu options [18]. Independent restaurants are an ideal setting in which to develop evidence-based approaches given the greater ability to work with those who will implement the changes as compared with chain restaurants [19]. A recent case study conducted in Texas with 16 independent restaurants determined that independent restaurants, and Latino restaurants in particular, are receptive to instituting menu changes for children’s menu items [20].

### 1.3. Present Study

The Kids’ Choice Restaurant Program (KCRP) adds to this research by describing the implementation and short-term efficacy of two approaches for promoting the sale of new healthy child menu items in independent restaurants. The study design allowed us to determine whether it was necessary to invest the additional time and resources to generate customer demand (through in-restaurant marketing and employee trainings in the menu-plus condition) versus simply making the healthy menu items available (as per menu-only condition) to achieve changes in sales of new and existing child menu items, both in terms of weekly sales in dollars and units sold. In their systematic review, Hillier-Brown et al suggest that more intrusive interventions that restrict or guide choice have a more positive impact on customers’ ordering when compared with information-only campaigns [14]. Thus, it was hypothesized that restaurants in the menu-plus condition would generate more dollars per week/more units sold from new healthy child menu items as compared to the menu-only condition during the eight-week intervention period and four-week post-intervention period (i.e., 12 weeks total). A secondary aim examined whether the addition of new healthy child menu items was effective at altering the sales of existing child menu items (weekly sales in dollars and units sold), where available, over the entire 16-week evaluation period, including a four-week baseline period. Process evaluation aims assessed implementation metrics, including feasibility and intervention fidelity.

## 2. Materials and Methods 

### 2.1. Study Design and Setting

KCRP was a cluster randomized trial, with pair-matched independent restaurants randomized to a menu-only versus a menu-plus intervention condition. Weekly sales data were collected from restaurant managers/owners to determine whether the interventions resulted in differential sales of new healthy child menu items, addressing our primary aim. A secondary aim examined whether the interventions resulted in a differential decrease in sales of existing child menu items. Intervention logs captured intervention implementation, addressing the process evaluation aim. Research evaluation staff also conducted unobtrusive observations and interviews with dining parties (not discussed in this paper). All of the research activities were conducted according to the guidelines laid down in the Declaration of Helsinki and the Institutional Review Board of San Diego State University approved all procedures involving human participants (IRB protocol #2100098).

The study occurred in San Diego County, CA between June and November 2015. This county had approximately 3.2 million residents with a median household income of $64,309 annually [21]. A third of San Diego adult residents identify as Latino; among children, this number is higher (46%).

### 2.2. Restaurant and Manager Recruitment and Allocation to Condition

**Figure 1:**
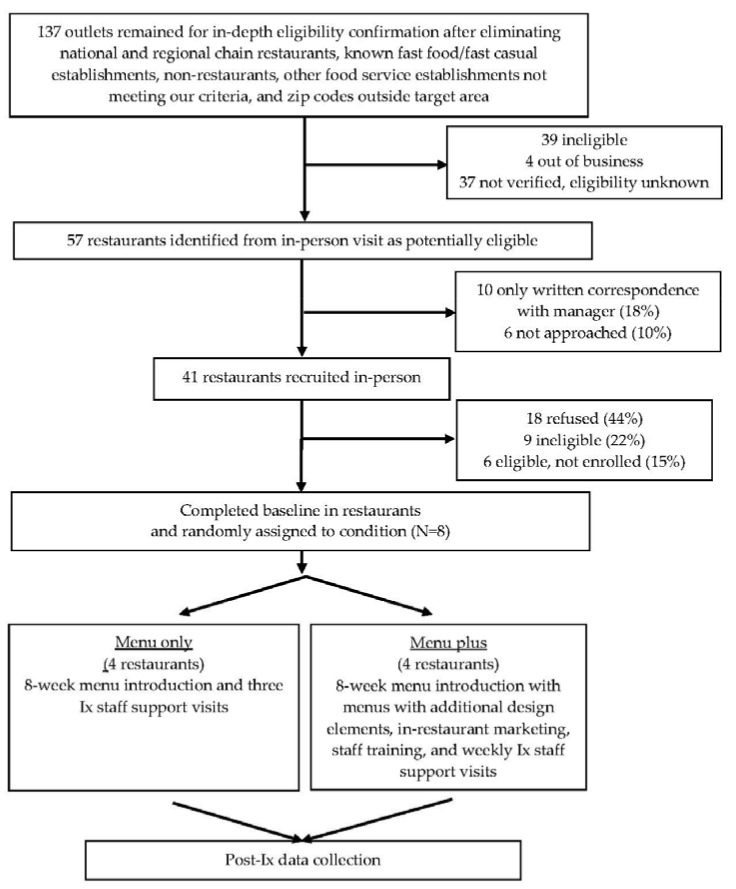
CONSORT figure for Kids’ Choice Restaurant Program (KCRP).

A detailed description of the restaurant enumeration process is available elsewhere [11]. Briefly, 12,759 food permit holders were identified using February 2014 San Diego County Department of Environmental Health food permits. Using internet searches, and phone calls where needed, the list was reduced to 532 by removing: restaurants out of business (*n* = 64); restaurants not meeting our inclusion criteria including known chain, fast food, and specialty-food (e.g., ice cream shop, soul) restaurants, as well as bars and other adult-only venues (*n* = 2834); non-restaurants (e.g., retail stores, amusement parks; *n* = 5081); duplicate entries (*n* = 3); and, by focusing on ZIP codes within a 5-mile radius of study offices (up to 10 miles in areas with a Latino population of at least 29% using 2010 U.S. Census data) (*n* = 4245). From among these 532 restaurants and six additional ones found through ground-truthing (e.g., new restaurants in out-of-business locations), 320 additional restaurants were excluded given that the type of food offered did not meet our inclusion criteria. From among the remaining 218, 81 were identified as potentially ineligible for other reasons (e.g., open fewer evenings), but requiring further verification prior to rejection for ineligibility or to enrollment, thus they were wait-listed. The remaining 137 restaurants were identified as requiring further assessment of study eligibility (see Figure 1). Restaurants were eligible if they were independently-owned, sit-down restaurants offering Latino (e.g., Mexican) or “American” food, such as hamburgers and other types of sandwiches, salads, BBQ, “diner” food, etc. Type of food was limited to minimize meal sharing (e.g., pizza at an Italian restaurant; family style meals at Chinese restaurants), which can result in fewer individual orders placed for/by children. The restaurant had to be willing and able to provide weekly sales data reports given our primary aim. In addition, to minimize sources of variance, the restaurants had to have 20 or more tables seating two or more individuals.

A trained bilingual (English/Spanish) research staff member visited restaurants selected for further assessment of eligibility and potential recruitment; the manager/owner was approached using an IRB-approved script. The restaurant manager/owner was informed of the study, including the requirement to be randomly assigned to one of two conditions, the level of commitment required in each condition, the timeline, and incentives. The latter included $300 for restaurant participation and $20 for completing each of two manager interviews (baseline and 12–13 weeks post-baseline). If the restaurant was eligible and the manager/owner was interested, the manager was screened for eligibility using an IRB-approved script and screening form. Restaurant managers/owners were eligible if they were at least 21 years old, had worked at least 20 h/week for at least the past four months for the participating restaurant, and planned to remain managing the restaurant for at least the next six months. In addition, he/she had to have decision-making authority over menu items and marketing strategies, and could not be employed at another participating restaurant to minimize potential cross-contamination. If eligible and agreed to study requirements, the restaurant manager/owner signed a letter of agreement and an informed consent form. The principal investigator and institutional official then signed the letter.

To minimize sources of variance across conditions over several waves of recruitment of restaurants from June to November 2015, prior to randomization, restaurants were pair-matched on the availability of an existing child menu (yes or no). In addition, with one exception, they were matched on one of two categories of size based on seating capacity (<100 vs. ≥100). The exception involved one set of pair-matched restaurants, both without a child menu but of different sizes. Cross-contamination was minimized by ensuring that the pair-matched restaurants were at least one-mile distance from each other. Once a pair was identified, the project biostatistician randomly assigned each restaurant to either the menu-only or menu-plus condition, and the intervention planning and implementation phase for the pair commenced. The overall timeline and restaurant involvement occurred as follows: weeks 1–2 introduction, paper work; weeks 3–6 baseline data collection and intervention planning; weeks 7–14 intervention implementation and process and outcome data collection ongoing; and, weeks 15–18 post-intervention process and outcome data collection.

### 2.3. Intervention Description

#### 2.3.1. Theoretical Framework

The Socio-Ecological Framework provided the overall theoretical foundation for the study given that it acknowledges multiple sources of influence on health behaviours and health outcomes, including social and physical environmental factors [22]. Intervention components were further informed by the integration of behavioural, organizational, and structural change theories [23,24,25]. For example, consistent with the concept of behavioural economics to automatize the selection of healthier versus less healthy menu items [26], and because of the importance of offering choices and being flexible [27], our child meals included a healthy side dish as a default and sometimes a healthy beverage (i.e., 1% or skim milk; 100% juice, water). Additional details on intervention development and formative research findings can be found at Castro et al. [28] and Ayala et al. [11].

#### 2.3.2. Intervention Conditions 

In both the menu-only and menu-plus conditions, restaurants were asked to introduce new healthy child menu items as guided by established nutritional criteria [29]; an entire meal (entrée, side, and drink) should be ≤600 calories and contain no more than 50% of calories from fat. This was a modification of the National Restaurant Association’s Kids LiveWell criterion of 35% fat given our goals for all of the new menu items to meet these criteria (vs. only one menu option), and to help gain buy-in and feasibility for the independent restaurants. Interventionists and restaurant managers/owners worked together to develop the new items focusing on the following four criteria: (a) kid-sized portions; (b) no fried foods; (c) fruit- or vegetable-based sides; and, (d) offering healthy beverages and no sugar-sweetened beverages. Menu planning started from a standard menu that was developed by the research team that included the recipes for new healthy child menu items and their nutritional analyses. During this initial meeting, standard healthy child menu items were presented to the manager and the team discussed which were feasible and appropriate to include in that restaurant’s menu and what was not necessary to change. For example, some chose to use their existing canned marinara sauce instead of making our from-scratch recipe, and it fit the nutritional requirements. For menu-plus restaurants, if they had an alternative item that they wanted to be considered for the new healthy child menu, they could modify an existing menu item (e.g., adult item modified for kids) or suggest a brand new item. Menu-only restaurants had limited flexibility with this. Ultimately, restaurants in both conditions provided the recipes to the study team who then ran the nutritional analyses on all of the newly proposed healthy child menu items. Nutritional information for each recipe was evaluated by entering the recipe into Food Processor Nutrition Analysis Software [30]. If the item had potential to meet our criteria for inclusion, we worked with the managers/owners to make it work (e.g., adjust 80/20 ground meat to 85/15 to get fat and calorie count down on a burger). If it did not work (e.g., pizza), we let them know it could not be included and worked together on alternatives. 

Following selection of menu items with restaurant management, specifically at least five main dishes, at least three healthy side dishes, and two healthy beverages, a research intervention staff member met with the kitchen manager/chef on the preparation and plating of these new healthy child menu items. A key focus of these meetings was on plating for children; they were encouraged to use smaller plates to retain a pleasing ratio of food to “white-space” [31]. Kitchen managers/chefs then trained the kitchen staff on the preparation and plating of the new menu items. Photographs of the plated items and recipes were provided to the kitchen staff at each restaurant. New child menus were printed in color and laminated; they included the restaurant and project logos, prices, and what the price included (e.g., with or without a beverage). Managers/owners were encouraged to set an “all-in-one” price and to be consistent with the pricing of existing child menu items. Consistent with previous efforts [26,32], restaurants with an existing child menu were encouraged but not required to remove existing child menu items from their printed menus and to stop offering them to customers. 

The menu-only restaurants presented these new healthy child menu items in a printed menu with new menu items presented in a randomly determined order and using few design elements. The menu-plus restaurants implemented an enhanced printed child menu and a healthy menu campaign, including table tents and signage, and up to two 15-min trainings for wait staff and one 15-min training for kitchen staff to promote the new healthy child menu items. Printed child menus included copy and design elements that influence ordering behaviour, such as placement of new menu items in specific locations on the menu (e.g., upper left hand corner), strategic formatting techniques (e.g., boxing menu items), and the use of catchy names and phrases [33]. Promotional materials were designed to appeal to children and adults based on our research [11,28,34,35], and those of others demonstrating the reciprocal influence of children and parents’ health behaviours outside the home [36]. All of the restaurant wait staff, including hosts and bussers, were invited to participate in a general 15-min training, providing an overview of the intervention, as well as customer service and suggestive selling techniques to promote the new healthy child menu items. A subset of the wait staff attended an advanced 15-min training focused on customer service strategies to use with different types of customers, again with the aim of promoting the healthy child menu items. Our planned reach was 50% given evidence of potential feasibility from our formative work [11] and previous studies with similar features in grocery stores [37]. The kitchen staff training served as an introduction to the program and focused on the new menu items and their appropriate portion sizes. 

#### 2.3.3. Implementation

In both conditions, the interventions were implemented over a concurrent eight-week period in pair-matched restaurants. The interventions that were directed to customers were initiated once all baseline data were collected, menus were finalized, and most of the trainings were completed. For each pair-matched restaurant, the interventions started on the same day. Printed menus were distributed to dining parties consistent with restaurant practices. After the new healthy child menus were introduced (and the healthy menu campaign in the menu-plus intervention condition started), a research intervention staff member conducted on-site support visits to help restaurant managers/owners to address any issues that arose with intervention implementation. Visits occurred three times over the eight-week intervention period, with menu-only restaurants, and with one exception, weekly with menu-plus restaurants. 

### 2.4. Evaluation Protocol and Measures

A mixed-methods approach was used to obtain process and outcome evaluation data. This included intervention implementation and short-term efficacy of the intervention on sales (weekly sales in dollars and units sold) of new healthy child menu items and existing child menu items.

#### 2.4.1. Process Evaluation-Intervention Implementation 

Our process evaluation aims were focused on determining the feasibility and potential fidelity of delivering these two interventions according to the planned dose (see Section 3). In both of the conditions, the research intervention staff member monitored implementation during the on-site support visits. The staff member documented the number of new child menus available, as well as table tents and promotional signage that was visible in the menu-plus condition. Training implementation was assessed in two ways: dose delivered, captured as minutes of training time delivered to each restaurant by type of training, and dose received, captured as % of employees trained and % of topic fully covered in the trainings.

#### 2.4.2. Short-Term Efficacy-Sales of New and Existing Child Menu Items

The primary outcome was weekly sales in dollars of new healthy child menu items; our secondary outcome was weekly sales in dollars of existing child menu items. In addition, we examined the number of units sold of each. Only sales of child meals (entrée and side; five of eight restaurants also included drink) were included; a la carte purchases of child sides or drinks were not included in these analyses due to the limitations in, and variation between, the sales data reports restaurants provided.

Weekly detailed sales data were collected from the restaurants during the baseline period for existing child menu items, and during the eight-week intervention period and the four-week post-intervention periods for the new and existing child menu items. Study eligibility required that restaurants could generate weekly sales reports containing, at minimum, total overall sales of foods and beverages and by item sales of individual child foods and beverages, including the new healthy child menu items. In addition, all of the restaurants were required to add the new healthy child menu items to their register systems prior to the implementation of the new menu.

All of the restaurants were able to provide sales in dollars and units. Project staff collected printed sales reports during scheduled visits for other project activities. Two restaurants elected to email excel reports that were generated by their register systems directly to the project. One restaurant refused to provide full sales reports and instead provided only total restaurant sales and manually generated reports of individual sales of child menu items. Sales data were coded by menu item, with each menu item receiving a unique code, and included the sales in dollars and units sold for each item. Items such as kitchen rental and catering, as well as alcoholic beverages that were listed on sales reports, were excluded from total restaurant sales.

#### 2.4.3. Manager Interview

Restaurant managers/owners were interviewed by a trained evaluator blinded to study condition at baseline and again at post-intervention (latter not presented here). Manager interviews assessed descriptive characteristics of the restaurants, managers, employees, and customers.

### 2.5. Statistical Analyses

Analyses were carried out according to the intention-to-treat rule. The unit of analysis was weekly sales in dollars and units sold by restaurants. Given that these data were nested within each restaurant, we used the linear mixed model approach to adjust for the restaurant clustering in the random effect. We also tested the time by condition interaction to explore whether the trends in the outcomes were different across the two conditions. Baseline sales in dollars and units sold were controlled for in analyses. Summer month indicator was also included in the fixed portion of the model to adjust for seasonality effects. Funding and time limits led to a small sample size, thus no other characteristics were adjusted in our models. Seasonality adjusted weekly sales in dollars and units sold were computed and graphed; however, these figures were very similar to the raw mean trends. Thus, raw mean trends are presented to reflect the actual sales in dollars and units. The multilevel models were fitted using SAS PROC MIXED (SAS Institute, Inc., Cary, NC, USA).

## 3. Results

### 3.1. Recruitment and Retention of Restaurants

Figure 1 depicts our CONSORT [38] figure. Refusal to participate among potentially eligible restaurants was moderately high at 44%, not unlike previous efforts (e.g., 48%, [20]). The low ineligibility rate was partly attributable to an extensive screening process that removed restaurants early in the process. Given our timeline, we screened, and in some cases approached, additional restaurants in case some did not agreed to participate, as depicted in Figure 1. The target sample of eight pair-matched restaurants was recruited and randomly assigned to condition. Six of eight restaurants had an existing child menu.

### 3.2. Restaurant Characteristics

Table 1 provides baseline restaurant characteristics by condition based on manager report and restaurant audits. Restaurants were similar across conditions, in part, given the matching design feature. The only differences that were observed between conditions was number of tables and overall sales with menu-only restaurants reporting more tables and fewer sales (though a larger range), and menu-plus restaurants reporting fewer tables and more sales.

### 3.3. Intervention Implementation

**Figure 2:**
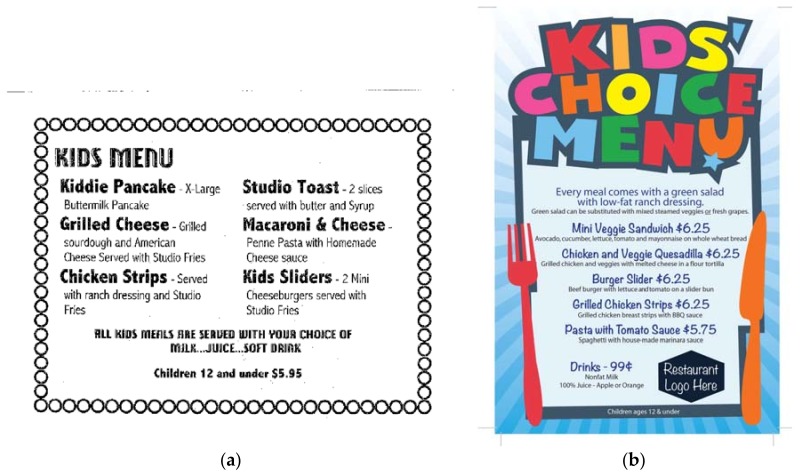

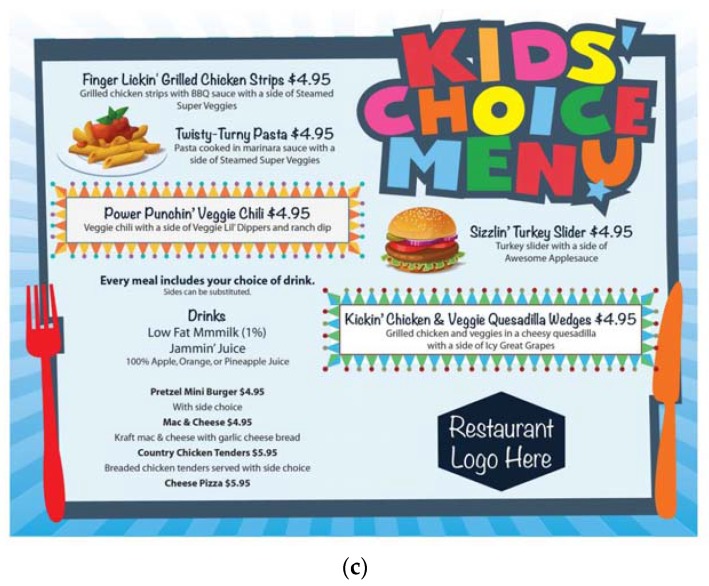
Example kids menus. (**a**) Original menu; (**b**) New menu from Menu-only condition; and, (**c**) New menu from Menu-plus condition.

Table 2 and Table 3 present intervention implementation in the two conditions, including price differences between existing and new healthy child menu items across conditions in Table 2, and the planned dose, dose delivered, and received in Table 3. Figure 2a,b depict examples of old and new menus from among restaurants in the menu-only condition. Figure 2c provides an example of a new healthy child menu in the menu-plus condition. All eight restaurants agreed to offer at least five new healthy entrees with healthy side dishes and healthy beverages not to exceed 600 calories or 50% of calories from fat for the meal and promote these items, at minimum, using a new printed menu. Restaurants were not required to remove any of the existing menu items, although they were encouraged to do so. Only one restaurant in any condition offered a side other than French fries. The most common existing beverages that were noted on the child menu were non-specific milk, juice, soda, or simply “drink”, and in most cases, this was included with the child meal. The price for existing and new healthy child menu items were fairly similar within condition, but different across conditions. In the menu-only condition, the cost was less but not when considering that one restaurant did not include the beverage with the new healthy child menu, but did include it with the existing child menu (see footnote). In the menu-plus condition, the healthy items were priced slightly lower than the existing child menu items.

Each restaurant received the number of menus that they reported needing (median supplied menus = 23; range 16–35), and the menus were mostly present at the visits. In the menu-plus condition, promotional signage was always present, but on average, only 65% of table tents were visible at the visits. Kitchen staff training at two of the four menu-plus restaurants was integrated with the kitchen manager/chef meetings, thus the duration was extended beyond the planned dose of 15 min. In these restaurants, the manager/owner invited all kitchen staff to attend the training together with the kitchen manager/chef. This allowed for consolidation of the training into one visit with the kitchen team instead of kitchen managers/chefs later training the rest of the kitchen staff on preparation and plating. Overall, in the menu-plus condition, approximately 50% of kitchen staff received the training. Wait staff training, both the general and advanced, was delivered as intended with most of the topics covered. However, less than 50% of the wait staff received the general training, and among those eligible to attend the advanced training, slightly over half received this training. Importantly, additional restaurant staff attended these trainings, including the manager/owner and bartenders. One menu-plus manager/owner was non-compliant with wait-staff trainings and displaying of table tents.

### 3.4. Primary Outcome Evaluation: Short-Term Efficacy on Sales of New Healthy Child Menu Items

**Figure 3:**
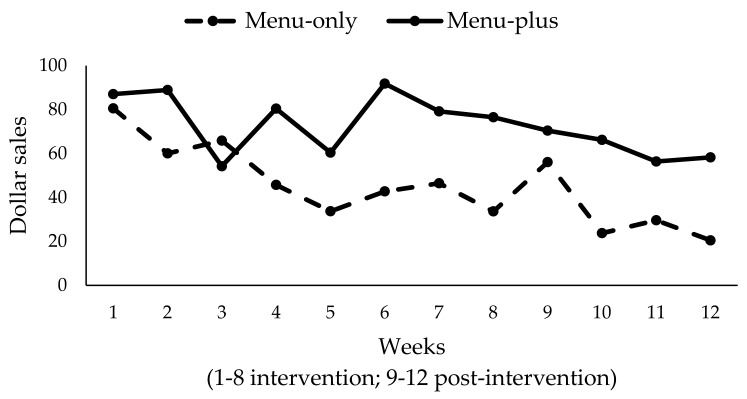
Total weekly $ sales of new healthy child menu items by condition (*N* = 8).

Baseline weeks were excluded from these analyses given the introduction of new healthy child menu items with the start of the intervention period, As such, weeks are numbered starting with intervention implementation as week 1. Analyses controlled for total restaurant sales in dollars at baseline. Results showed a significant downward trend for weekly dollar sales of new healthy child menu items (*β* = −3.20; SE = 1.08; *p* = 0.004) in both of the conditions (Figure 3), but not a significant time-by-condition interaction. Limiting our analysis to the eight intervention weeks only, we found that the main effect for time was not significant. This suggests that the significant decline for weekly dollar sales occurred in the post-intervention period in both conditions. Similarly, a significant downward trend for units of new healthy child menu items sold was also found (*β* = −0.58; SE = 0.18; *p* = 0.002), with no significant difference by condition (figure not shown). As before, limiting our analyses to the intervention period demonstrated that the downward trend was driven by declines in units sold during the post-intervention period in both conditions. Finally, we conducted sensitivity analyses excluding the menu-plus restaurant that was non-compliant with key intervention components (see Section 3.3) and found significantly higher sales of healthy child menu items (in terms of both dollars and units) in menu-plus restaurants compared to menu-only restaurants (weekly sales in dollars: *β* = 52.81; SE = 12.96; *p* = 0.0001; units sold: *β* = 9.0; SE = 2.67; *p* = 0.001). Nevertheless, the significant downward trend remained present over time in both conditions (weekly sales in dollars: *β* = −3.29; SE = 1.18; *p* = 0.007; units sold: *β* = −0.61; SE = 0.20; *p* = 0.003).

### 3.5. Secondary Outcome Evaluation: Short-Term Efficacy on Sales of Existing Child Menu Items 

**Figure 4:**
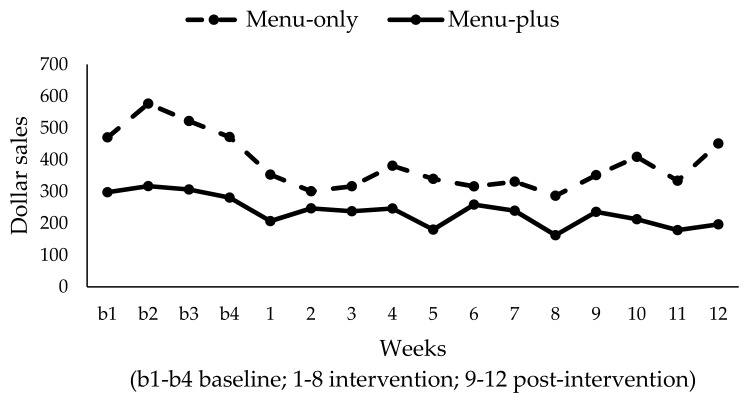
Total weekly sales in dollars of existing child menu items by condition (*N* = 6).

Among restaurants with an existing child menu (*n* = 6) and controlling for baseline sales in dollars, a significant time by condition interaction was found for weekly dollar sales of existing child menu items (*β* = −9.68; SE = 4.20; *p* = 0.025). As indicated in Figure 4, the menu-only condition had an upward dollar sales trend during the post-intervention period, whereas the menu-plus condition showed a slight downward trend during the same period. We further tested the time by condition interaction for only the intervention period and no significant interaction was found. This suggests that the difference in sales occurred in the post-intervention period. Similarly, for units of existing child menu items sold and controlling for baseline existing child menu units sold, a significant time by condition interaction was observed (*β* = −1.68; SE = 0.72; *p* = 0.022), with findings similar to those of weekly sales in dollars (figure not shown). As before, limiting our analyses to the intervention period demonstrated that the differences in units sold occurred during the post-intervention period. In analyses excluding the non-compliant menu-plus restaurant, the observed interactions remained significant for units but not for weekly sales in dollars (weekly sales in dollars: *β* = −9.29; SE = 5.00; *p* = 0.067; units sold: *β* = −1.65; SE = 0.82; *p* ≤ 0.05).

**Figure 5:**
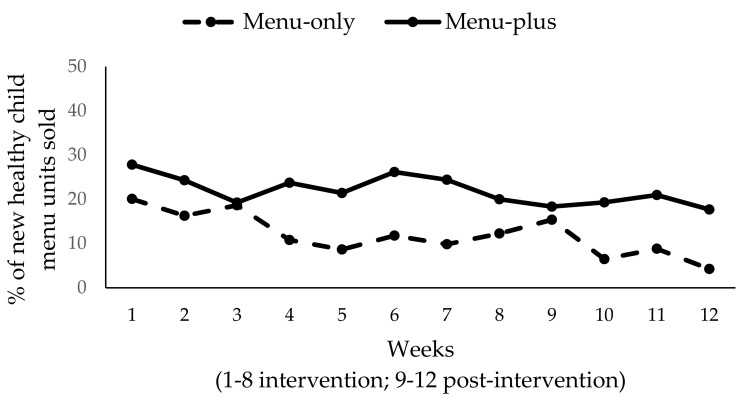
% of healthy child menu items (units) sold by condition (*N* = 6).

In the menu-only condition, total sales of existing child menu items were, on average, $510/week. In the menu-plus condition, the total sales of existing child menu items was, on average, $301/week. When comparing these data to the first four weeks of the intervention period, we observed that weekly sales from existing child menu items in the menu-only condition were $338 as compared with $70 for new healthy child menu items. In the menu-plus condition, weekly sales from existing child menu items were $235 when compared with $69 for new healthy child menu items. This suggests that of the total child menu sales, 17% of sales were for new healthy child menu items in the menu-only condition as compared with 23% in the menu-plus condition. Displayed in terms of units, Figure 5 shows the % of all the child menu item units sold were new healthy menu items among restaurants with an existing child menu. The graph shows that the percent of healthy child menu items sold was higher for the menu-plus condition for most of the intervention and post-intervention weeks, with a relatively stable trend of 20% across time. 

## 4. Discussion

In this study, two intervention approaches were tested in eight independent, sit-down restaurants offering American or Latino menu items to test whether introducing healthy child menu items would affect the meals that were purchased for/by children. We worked with all of the restaurants to create healthy child menu items with meals, including a healthy entrée, a fruit- or vegetable-based healthy side dish, and for five of eight restaurants, the healthy beverage (low fat or nonfat milk, 100% juice, water) was included. Half of the restaurants received just the new menu (i.e., menu-only). The other half also received a strategically designed new healthy child menu, marketing materials, and training to support implementation, including trainings for their wait staff to encourage customers with children to order the new healthy child menu items (i.e., menu-plus). In the menu-plus restaurants, about half of the intended employees received the trainings and all but one of the restaurants were supportive of displaying the marketing materials. Challenges with engaging busy business members have been identified in previous restaurant-based interventions [20]. We evaluated the sales of both new and existing child menu items over the same period in both of the conditions. Sales of the new healthy child menu items occurred immediately in both of the conditions, but went down during the post-intervention period, along with overall child menu sales. Also, during the post-intervention period, sales of the existing child menu items went down in the menu-plus condition versus going up in the menu-only condition. When one non-compliant restaurant was excluded from the analyses, sales of the healthy child menu items were significantly higher in menu-plus versus menu-only restaurants, suggesting that when the menu-plus intervention was delivered with fidelity, the additional components made a positive impact on sales. The findings underline the importance of fidelity to intervention components when drawing causal inferences. Additional research utilizing multi-component interventions to promote healthier child menu items is warranted. In recent years, recommendations for “performance standards” for restaurants were developed by an expert panel [18]. This study provides preliminary evidence supporting the use of the recommended practices and demonstrating that implementing more of these practices (i.e., menu-plus vs. menu-only) may yield larger improvements in ordering.

### 4.1. Strengths and Limitations

This study and its findings contribute to the literature on AFH consumption by studying the sales of new healthy child menu items and existing child menu items in response to two intervention approaches in independent restaurants, a context that is pervasive in high risk communities, yet is understudied in research. A major strength of this study is the rigorous study design as few randomized controlled trials have been conducted examining the sales of healthy child menu items [7,14]. The healthy child menu items were determined through collaboration with restaurant managers, thus allowing for restaurants to leverage ingredients that are used in other dishes, while reducing food waste and staff burden related to food preparation. The trainings were open to other restaurant employees, including the manager/owner, which likely helped with implementation.

Despite these strengths, a number of limitations emerged. First, differences in restaurants’ register systems made data collection and processing challenging, time-consuming, and in some cases, limited the data available for analyses. For example, child meals were often identified on sales reports by the entrée name only; thus, we could not examine variations in meal composition. Unhealthy add-ons and substitutions (i.e., asking to remove vegetables from quesadilla) to a new healthy child meal can negate the impact of the new menu item [39]. Second, restaurants were reluctant to remove unhealthy child menu items, such as grilled cheese sandwiches and fried chicken tenders from their menus; workarounds such as those enacted by other researchers and practitioners may be warranted (e.g., continued encouragement to remove from printed menu but make available upon request; [20,26,40]). Similarly, some restaurants that already had child menus priced the new healthy child menu items at a price point that was slightly higher than existing child menu items, citing overall rising costs and the need to modify prices. This may have disincentivized their purchase by customers; however, importantly, overall the healthier menu items were similar or cheaper than existing child menu items within condition. Third, one set of pair-matched restaurants differed in size given our interest in matching on availability of child menu; this was controlled for in analyses. Fourth, funding constraints limited the amount of time that kitchen and wait staff had to familiarize themselves with the new healthy child menu items before they were offered to customers; time for engagement can affect participation as was evident in the one restaurant that was non-compliant. This same constraint limited the number of restaurants that were recruited to participate, thus reducing statistical power, as well as the length of the intervention period to a timeframe that may have been too short for customers’ behaviours to be impacted sufficiently to achieve larger sales changes. Notwithstanding, as the field moves toward designing for dissemination, critical considerations from restaurant managers is essential [19,27]. Finally, it is possible that children ate at more than one participating restaurant during the study period as the interventions were implemented at the same time in pair-matched restaurants. However, it is unlikely that their experience in one restaurant would generalize to another to a degree that would contaminate results at the restaurant level.

### 4.2. Implications

Independent restaurants are willing to modify what they offer to children, and in the short term, customers appear to continue to buy these new healthy menu items for their children. Future research in this area should consider increasing the length of the evaluation period to examine the extent to which the menus and sales changes are sustained. Future research should also consider methods for encouraging the elimination of less healthy existing child menu items from the restaurant’s offering. Our formative research suggested that most children have already decided what to order before they arrive at the restaurant [28]; thus, additional promotions external to the in-restaurant environment, and highlighting that new menu items are available, may be needed to achieve changes in sales. Research on the amount of employee training needed to be meaningfully related to changes in customers’ ordering behaviors is needed to inform dissemination and implementation efforts. Finally, future studies may want to invest in new register systems and/or training for participating restaurants, in addition to more extensive training of wait staff on how to enter items of interest in when keying in an order, to obtain more consistent and useful data on side dishes and beverages for analysis purposes.

## 5. Conclusions

Results suggest that the inclusion of additional in-restaurant marketing and employee trainings modestly impacted the sales of healthy child menu items over and above changes observed when menu items were simply made available. Both of the conditions resulted in sales of the new menu items; however, refined intervention approaches may be more effective in creating larger and lasting change. For example, by design, the new menus made no mention of healthy, though evidence obtained during the study suggested that customers who are parents/caregivers may be more eager and open to seeing “healthy” options being promoted for their children than for themselves [28]. Thus, future studies may want to consider how best to promote the new options as healthy alternatives given strong evidence that healthy is often equated to less tasty [41]. Given the ubiquity of independent restaurants and their importance to many families’ diets, more research is needed with independent restaurants. 

## Figures and Tables

**Table 1:** Kids’ Choice Restaurant Program baseline restaurant characteristics.

Restaurant Characteristics	Overall	Menu-Only	Menu-Plus
	Mean (SD) or Median (Range) or % (*n*)		
Number of restaurants	*N* = 8	*n* = 4	*n* = 4
Mean years in operation	17.8 (15.0)	17.8 (20.5)	17.7 (10.3)
Mean number of full-time employees	12.9 (6.6)	11.8 (5.7)	14.0 (8.2)
Mean number of part-time employees	11.1 (11.5)	12.3 (14.1)	10.0 (10.4)
Mean number of tables	33.0 (7.2)	36.8 (4.5)	29.3 (7.9)
Restaurants’ marketing in English only	87.5% (7)	75.0% (3)	100% (4)
Restaurants with existing child menu	75.0% (6)	75.0% (3)	75.0% (3)
Median of manager reported average weekly sales	$21,500 ($8000–60,000)	$16,000 ($8000–60,000)	$23,750 ($13,461–32,000)

**Table 2:** Characteristics of existing child menu items (*n* = 6) and price of new healthy child menu items (*N* = 8).

Child Menu Characteristics	Menu-Only	Menu-Plus
Composition of existing menu (*n* = 6; 3 per condition)		
Offer healthy sides	33% (1)	33% (1)
Existing beverage included	66% (2)	66% (2)
Price (average)		
Existing child menu item ^1^	$6.67	$5.50
New healthy child menu item ^2^	$6.55 ^3^	$5.47

^1^ Average computed by summing the price of existing child menu items across restaurants (6 of 8) within condition, and then dividing by the total number of items; ^2^ Average computed by summing the price of new child menu items across all 8 restaurants within condition and then dividing by the total number of items; ^3^ Average price increases to $6.88 when accounting for beverage price in one restaurant that included beverage with existing child meals but did not include in new healthy child meals.

**Table 3:** Kids’ Choice Restaurant Program implementation.

Program Implementation	Planned Dose	Menu-Only (*n* = 4) Median (Range)	Menu-Plus (*n* = 4) Median (Range)
Number of healthy main entrees offered	5	5 (5–6)	5.5 (5–6)
Median % of supplied menus present across site visits per restaurant	100%	89.0% (70–92%)	95.0% (91–100%)
Median % of tables displaying table tents across site visits per restaurant	100%	NA	65% (0–95%)
Median number of promotional signs always displayed across site visits per restaurant	1	NA	1 (1–2)
Average minutes of kitchen manager/chef meetings	NA	59.5 (37–80)	62.0 (60–64) ^a^
Dose Delivered			
Average minutes of kitchen staff training	15	NA	33.0 (5–49) ^b^
Average % of topics covered in kitchen training ^c^	100%	NA	87.5% (88–100%)
Average minutes of general wait staff ^d^ training	15	NA	12.0 (0–19)
Average % of topics covered in general training ^c^	100%	NA	91.7% (83–92%)
Average minutes of advanced wait staff training	15	NA	11.0 (0–19)
Average % of topics covered in advanced training ^c^	100%	NA	91.7% (83–92%)
Dose Received			
% of kitchen staff received kitchen training	100%	NA	47.2% (29–50%)
% of wait staff received general training	100%	NA	38.5% (0–78%)
% of wait staff received advanced training	50% ^e^	NA	56.0% (0–86%)

NA = not applicable. ^a^ Meetings with kitchen managers/chefs only in two of the four restaurants; ^b^ Kitchen staff trainings occurred with kitchen manager/chef meeting in two of the four restaurants; ^c^ Interventionists logged delivery of trainings and noted information not presented or activities not completed; ^d^ Wait staff included servers, hosts/hostesses, and bussers; ^e^ Of those who received the general training.
